# The Investigation of Percutaneous Tibial Nerve Stimulation (PTNS) as a Minimally Invasive, Non-Surgical, Non-Hormonal Treatment for Overactive Bladder Symptoms

**DOI:** 10.3390/jcm12103490

**Published:** 2023-05-16

**Authors:** Connor McPhail, Robert Carey, Sidharth Nambiar, Nadia Willison, Saghi Bahadori, Pouria Aryan, Tran Nguyen, Fariba Behnia-Willison

**Affiliations:** 1 FBW Gynaecology Plus, Adelaide 5035, Australia; 2 Department of Obstetrics & Gynaecology, Flinders Medical Centre, Bedford Park 5042, Australia; 3 Flinders University, Adelaide 5042, Australia; 4 Adelaide Medical School, University of Adelaide, Adelaide 5005, Australia; 5 School of Electrical & Electronic Engineering, University of Adelaide, Adelaide 5005, Australia

**Keywords:** PTNS, overactive bladder, urge incontinence, minimally invasive, non-surgical

## Abstract

Background: Overactive bladder (OAB) syndrome affects 10–15% of women, severely impacting their quality of life. First-line treatments include behavioural and physical therapy, and second-line medical treatments include medications such as vaginal oestrogen, anticholinergic medications, and ß3-adrenergic agonists—with potential adverse side effects including dizziness, constipation, and delirium, particularly affecting elderly populations. Third-line treatments include more invasive measures, including intradetrusor botulinum injections or sacral nerve modulation, with percutaneous tibial nerve stimulation (PTNS) being a potential alternative treatment. Aims: The aim of this study was to explore the long-term efficacy of PTNS treatment for OAB in an Australian cohort. Materials and Methods: This is a prospective cohort study. Patients underwent Phase 1 treatment, whereby women received PTNS treatment once per week for 12 weeks. Following Phase 1, women entered Phase 2, whereby they received 12 PTNS treatments over 6 months. Their response to treatment was measured by obtaining data before and after each phase using ICIQ-OAB and the Australian Pelvic Floor Questionnaire (APFQ). Results: Phase 1 included 166 women, with 51 completing Phase 2. There was a statistically significant reduction in urinary urgency (29.8%), nocturia (29.8%), incontinence (31.0%), and frequency (33.8%) compared to the baseline. Patients who completed Phase 2 also showed a statistically significant reduction in urinary frequency (56.5%). Conclusions: Overall, the results from this study are positive and support that PTNS is a minimally invasive, non-surgical, non-hormonal, and effective treatment for OAB. These results suggest that PTNS may be a second-line treatment for patients with OAB not responding to conservative management or for patients aiming to avoid surgical approaches.

## 1. Introduction

Overactive bladder (OAB) is a clinical condition which can be accompanied by urinary frequency and/or nocturia OAB affects millions of people worldwide and is more common in women and older adults, but it can occur in people of all ages and both genders. The condition can have a significant impact on quality of life, including decreased productivity and increased absenteeism from work, as well as on social and emotional well-being OAB affects 10–15% of women across all age groups, with the prevalence increasing with age and potentially severely impacting women’s quality of life [1,2,3]. These women tend to engage in elaborate behaviours to hide and manage their condition, including limiting daily activities, restricting fluid intake, reducing exercise and social outings, and wearing incontinence pad [1,4].

The exact causes of OAB are not fully understood, but several factors have been identified as potential contributors. These include neurological disorders, such as Parkinson’s disease, bladder outlet obstruction, and detrusor overactivity (unusual muscle contractions in the bladder). Pelvic organ prolapse can also cause symptoms of OAB [5,6,7]. Some studies have also suggested that OAB may be linked to lifestyle factors, such as diet and fluid intake, and underlying medical conditions, such as urinary tract infections or interstitial cystitis [1,8]. In some cases, the cause of OAB may not be clear and it may be referred to as idiopathic OAB [9].

Treatments for OAB currently available can be classified as first-, second-, and third-line treatments. First-line treatments are behavioural modifications, for instance bladder re-training, which involves gradually increasing the time between voiding, or lifestyle changes, such as reducing fluid intake and avoiding bladder irritants, by excluding caffeinated drinks, optimising weight, smoking cessation and pelvic floor exercises [9,10].

Behavioural modifications are a common first-line treatment for OAB, but they may have limitations for some individuals, such as an inability to reduce fluid intake due to other comorbidities. Additionally, pelvic muscle exercises (also known as Kegel exercises) require consistent and regular effort, which may prove difficult for patients [11,12]. Behavioural modifications alone may not be enough to adequately manage OAB symptoms, and additional treatments such as medications or invasive procedures may be necessary [11,12,13]. 

Second-line treatment includes anticholinergic drugs, vaginal oestrogen, beta-3 agonists, and desmopressin [9,14]. Medications used to treat OAB include antimuscarinics, beta-3 agonists, and desmopressin. Antimuscarinics work by blocking the actions of a chemical called acetylcholine, which is involved in bladder contractions. Beta-3 agonists stimulate beta-3 receptors in the bladder, which can help reduce bladder contractions. Desmopressin is a hormone that helps reduce the amount of urine produced by the body. The most appropriate medication will depend on the individual and the severity of their OAB symptoms. 

However, like all medications, there may be risks associated with their use. Some common side effects of OAB medications include dry mouth, constipation, blurred vision, and headaches [9,11]. 

Current treatment regimens may have significant side effects. Anticholinergic drug therapy such as Darifenacin—marketed as Enablex^®^, Solifenacin—marketed as Vesicare^®^ or Oxybutynin, oral Oxybutynin—marketed as Ditropan^®^ are associated with dizziness, dry mouth, constipation, reflux, blurred vision, delirium, and cognitive impairment, particularly affecting elderly populations. Some medications may also cause an allergic reaction, and in rare cases, they can lead to more serious side effects such as an increased risk of urinary retention or urinary tract infections [15].

Third-line treatment includes intradetrusor injection of botulinum toxin (Botox) [16,17], percutaneous tibial nerve stimulation (PTNS) [18], and sacro-neuromodulation (SNM) implants Botulinum toxin (Botox) injection is a minimally invasive procedure used to treat OAB symptoms. Botox works by blocking the nerve signals that cause the bladder muscles to contract, resulting in relaxing of the muscles. This leads to an improvement in bladder control and a reduction in OAB symptoms.

The benefits of using Botox for OAB treatment include quick recovery time and minimal discomfort during the procedure [17,18]. The effects can last for several months, although the duration of relief may vary from person to person, and regular follow-up injections may be necessary to maintain the benefits Additionally, it may not be suitable for everyone, and a doctor should be consulted to determine if it is an appropriate treatment option for a particular individual. Despite these considerations, Botox injections have been shown to be an effective treatment option for many people with OAB symptoms. Botox injection for the treatment of OAB is generally considered safe, with a low risk of side effects However, some patients may experience temporary side effects, such as urinary tract infection, bladder perforation, and urinary retention. In rare cases, there may be long-term side effects, such as urinary incontinence or chronic pain 

SNM is a minimally invasive surgical procedure that involves the implantation of a small device to treat OAB. The device stimulates the sacral nerve, which is responsible for controlling the bladder muscles. This stimulation helps to regulate the bladder and reduce OAB symptoms, such as urinary urgency, frequency, and incontinence [19].

SNM is considered a relatively safe and well-tolerated treatment option for OAB [20,21]. The procedure involves the implantation of a small device under the skin, which is controlled by an external remote. The device can be adjusted or turned off if needed, and the procedure can be reversed if necessary. SNM has a relatively low risk of complications, and the majority of patients experience significant improvements in OAB symptoms, with minimal side effects [21].

Risks associated with SNM include infection, as with any surgical procedure, and pain or discomfort either at the site of the implant or where the leads are attached to the sacral nerve. Lead migration may also occur where the leads attached to the sacral nerve may move out of place, requiring revision surgery to correct. The SNM may rarely malfunction or stop working, requiring replacement or removal [22].

While SNM is effective in reducing OAB symptoms in many patients, some may continue to experience symptoms despite treatment. Due to the risks and complications of some treatments for OAB, PTNS has become more favourable, as it is a non-surgical, non-hormonal treatment for OAB. PTNS uses a neuromodulator to stimulate the tibial nerve, altering sacral nerve plexus neurotransmission, thereby indirectly modulating bladder function. The mechanism of how PTNS improves OAB is unknown, but hypothesised as increasing suppression of the overactive detrusor muscle by increasing inhibitory spinal interneuron function.

PTNS is a minimally invasive procedure that involves the placement of a small needle near the tibial nerve in the ankle, which is then connected to a stimulation device that delivers a mild electrical current. The goal of PTNS is to activate the tibial nerve and improve bladder control. Studies have shown that PTNS can be an effective treatment for OAB. In a systematic review and meta-analysis [23], PTNS was found to significantly improve bladder control and reduce urinary urgency and frequency compared to the control group. Additionally, PTNS has been shown to have a lasting effect, with patients reporting improvement in symptoms for one to three months after the procedure [23,24].

PTNS is generally well tolerated and has few side effects. The most common side effects include mild discomfort during the procedure, skin irritation at the needle site, and mild muscle spasms. Unlike other surgical treatments for OAB, PTNS is minimally invasive and does not require general anaesthesia This makes it a suitable option for patients who are not candidates for more invasive treatments, or who prefer to avoid surgery.

Despite its effectiveness, PTNS is not a cure for OAB and may not be suitable for everyone. It is important to discuss the risks and benefits of PTNS with a healthcare provider before deciding to undergo the procedure. Healthcare providers will consider factors such as the severity of OAB symptoms, the patient’s overall health, and any underlying medical conditions when determining whether PTNS is the best treatment option [23].

Current publications demonstrate PTNS to be effective in treating non-obstructive urinary retention, neurogenic bladder, chronic pelvic pain, painful bladder syndrome, voiding dysfunction, faecal incontinence, and OAB symptoms [18,23]. PTNS is not currently promoted as a mainstream second-line treatment for OAB despite the available favourable peer-reviewed evidence. 

A 2021 review paper considered more than two decades of publication in the literature [21] (Nitti, Patel et al. 2021), which suggested that “a stepwise approach to treatment through first-, second-, and third-line therapies is recommended, recognising this may not be appropriate for all patients” [21].

Ultimately, the best treatment for OAB will depend on the individual patient and may involve a combination of different approaches. It is important to work with a healthcare provider to determine the most appropriate treatment plan. With proper management, many people with OAB can achieve significant improvements in their symptoms and quality of life [3,14]. 

The current research study aims to explore the long-term efficacy of PTNS treatment for OAB in an Australian cohort.

## 2. Materials and Methods

### 2.1. PTNS Treatment

PTNS involves the patient sitting comfortably in a chair with one leg extended and barefoot raised on a footstool. A PTNS needle is inserted 3 cm superior and 2 cm posterior to the medial malleolus to approximate tibial nerve location as per the manufacturer’s protocol. The area is cleaned with 2% alcohol before electrode insertion and connected to a hand-held Endotherapeutics Urgent PC neuromodulation system via disposable electrode leads, with the neutral electrode (pad) attached to the patient’s lower limb with an adhesive patch. The practitioner tests for the correct placement of the electrode through a test function, causing an involuntary toe-twitch or curl if correctly placed. The applied voltage required for satisfactory nerve stimulation is then tailored to the individual, varying with proximity to tibial nerve location and the patient’s comfort. The treatment then includes 30 min of tibial nerve stimulation. 

### 2.2. Data Collection

Patient response to PTNS was measured using two validated questionnaires, the Australian Pelvic Floor Questionnaire (APFQ) and the International Consultation on Incontinence Questionnaire Overactive Bladder Module (ICIQ-OAB) at baseline and after each treatment phase at FBW Gynaecology Plus (Adelaide, South Australia), with the PTNS treatments conducted in an outpatient setting.

### 2.3. Study Methods

Inclusion criteria included patients with urinary urgency or incontinence and patients were excluded if previously proven to not have urodynamic evidence of detrusor overactivity. Phase 1 (introductory phase) patients underwent 12 treatments over the course of 12 weeks. The recruited patients were offered pharmacological, PTNS, and botox treatments, with urodynamic studies completed if botox was already attempted to demonstrate excessive detrusor activity. The patients underwent an ICIQ-OAB quality of life questionnaire before they started their first treatment and after they finished each phase of treatment. Once the patients had completed 12 weekly treatments in the first phase, they were reviewed by the gynaecologist in order to determine the efficacy of treatment for the individual and confirmation for continuing into Phase 2 (tapering phase). Phase 2 included five PTNS treatments over 12 weeks, with patients being eligible for Phase 2 if they had 50% or more improvement in OAB symptoms during Phase 1 as assessed by the treating gynaecologist. The patients then entered Phase 3 (maintenance therapy), designed to sustain the symptom improvement gained through Phase 1 and Phase 2 via monthly PTNS treatment indefinitely if they demonstrated clinical improvement from Phase 2.

The data analysis was completed with Microsoft Excel. A paired *t*-test was used for assessing symptom level after the PTNS session. Statistical significance was set as *p*-value < 0.05.

Urinary frequency refers to the need to urinate frequently or an increased number of times per day compared to normal. Urinary frequency was assessed as per the ICIQ-OAB scoring from 0 to 4 according to the number of daily micturition episodes (Appendix A). Urinary urgency is a sudden and strong desire to urinate that cannot be deferred or postponed. Urinary urgency was assessed as per the ICIQ-OAB scoring from 0 to 4, representing a subjective frequency of the number of times the patient had to rush to micturate (Appendix A). Nocturia is the need to awaken at night to urinate. Nocturia was assessed as per the ICIQ-OAB scoring from 0 to 4 (Appendix A). Urinary urge incontinence (UUI) is the involuntary loss of urine associated with a strong, sudden urge to urinate. UUI was assessed as per the ICIQ-OAB scoring from 0 to 4 (Appendix A).

## 3. Results

There were 166 patients who completed Phase 1, with 51 continuing to Phase 2, and 16 eligible for Phase 3. The mean age was 61 ± 16 years. Changes in ICIQ-OAB scores across urinary frequency, urgency, nocturia, and urge incontinence were used to assess symptom improvement. From these data, the results are as follows, with all analysed symptoms except urinary frequency at Phase 1 statistically significant (Table 1).

### 3.1. Urinary Frequency

Completion of Phase 1 (introductory phase) was associated with a 31% reduction in symptom severity (Table 1). Completion of Phase 2 was associated with a further reduction in symptom severity by 56% (Table 1).

### 3.2. Urinary Urgency

Completion of Phase 1 was associated with a 29.8% reduction in symptom severity (Table 1). Completion of Phase 2 was associated with a further reduction in symptom severity of 8% (Table 1). There was no significant difference between the results at the end of Phase 1 and Phase 2 (*p* = 0.39, paired *t*-test).

### 3.3. Nocturia

Completion of Phase 1 was associated with a 29.8% reduction in symptom severity (Table 1). Completion of Phase 2 was associated with a further reduction in symptom severity of 8% (Table 1). Symptom severity from the end of Phase 1 to the end of Phase 2 was not statistically significant (n = 51, *p* = 0.05, paired *t*-test).

### 3.4. Urinary Urge Incontinence

Completion of Phase 1 was associated with a 31.0% reduction in symptom severity (Table 1). Completion of Phase 2 was associated with a further reduction in symptom severity of 0.71% (Table 1). There was a trend towards reduced symptom severity from Phase 1 to 2.

**Figure 1:**
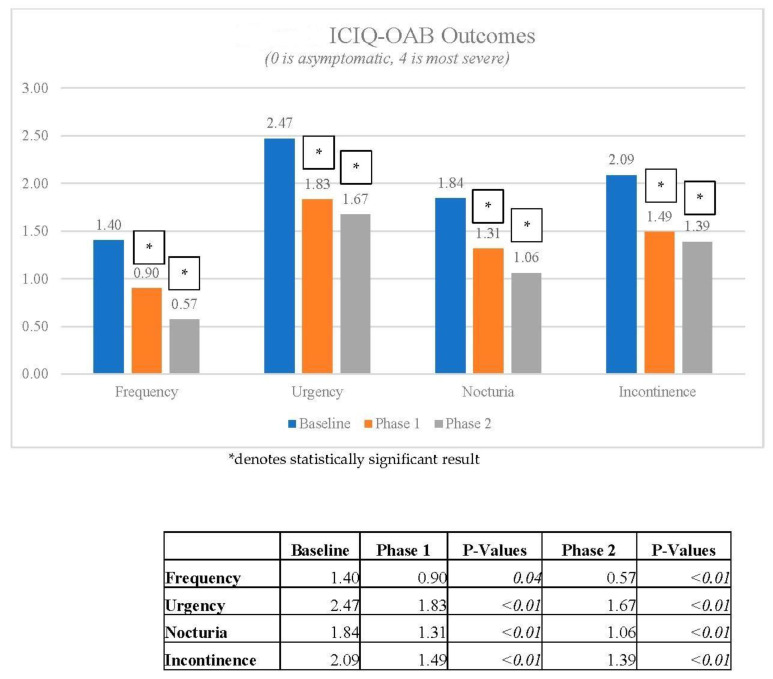
ICIQ-OAB Outcomes.

In Figure 1, ICIQ-OAB outcomes were statistically significant from baseline to Phase 1 and baseline to Phase 2, but there was no statistical change between Phases 1 and 2.

**Figure 2:**
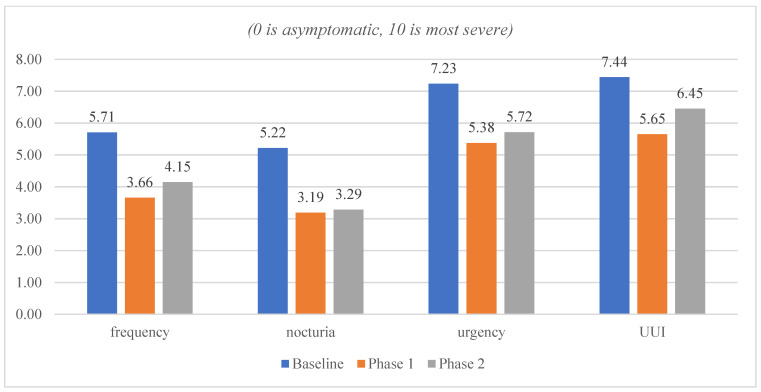
ICIQ-OAB Bothersome Level.

In Figure 2, the ICIQ-OAB bothersome level was measured on a scale of 0 to 10, with 0 being least bothersome and 10 being most bothersome, which improved from baseline to Phase 1 and remained stable in Phase 2.

## 4. Discussion

The aim of this study was to confirm the reduction in OAB symptom severity after PTNS treatment in an Australian setting. This expectation was based on results from previous literature [25,26,27]. In the current research study, the findings from Phase 1 followed the expected trend of significantly reducing the burden of disease in all four OAB symptoms.

Weekly PTNS sessions appeared to be effective in alleviating OAB symptoms, as seen in the literature, which had previously established the efficacy of PTNS treatments in non-Australian settings [25,26,27]. The above results demonstrated statistically significant short-term improvements in urinary frequency, urinary urgency, nocturia, and urinary urge incontinence. Long-term statistically significant improvements were also reported in urinary frequency and nocturia. Phase 2 aimed to maintain the therapeutic benefits established in Phase 1 but, interestingly, resulted in a further statistically significant reduction in urinary frequency, while the other three OAB symptoms showed a downward trend in severity. This additional therapeutic benefit of Phase 2 requires further evaluation due to a loss of follow-up between Phase 1 and Phase 2, as affected by COVID-19 restrictions. There were no reported adverse side effects of PTNS treatment, suggesting that PTNS is a safe and viable early treatment for OAB.

There were several challenges associated with this study. The first was the level of follow up in Phase 2 and 3 due to the COVID-19 pandemic. A larger Australian study on the long-term effects of PTNS is required to further support the current study’s findings and to further establish the additional therapeutic improvement observed after Phase 2. Long-term compliance with treatment regimens may be a potential difficulty associated with PTNS treatments, as frequent visits to a PTNS clinic involving at least 30 min of treatment may prove to be difficult for patients, especially in rural/remote areas or those with difficulty accessing PTNS clinics. Verbal feedback during the study period suggested that the sustained clinical improvements from Phase 2 meant that the travel and treatment times were not justifiable enough to encourage ongoing maintenance treatment. A brief period involved a gap payment for patients as this service was provided in a private clinic, decreasing the number of those continuing with the PTNS treatment and affecting follow-up. Micturition calendars were not used, as patients reported that when completing these during the initial investigation period of their bladder symptoms, the documents were cumbersome and they were reluctant to complete them subsequently, potentially affecting both quality of obtained data and long-term follow-up during the study. Initial questionnaires were double-sided, with some patients leaving the second-page questions unanswered due to this administration error, which was fixed during the study period, with improved answering rates after. The study design was also a prospective study without a comparison group, and so the improvement could be secondary to a placebo effect, although patients were not given access to previous questionnaires to reduce the risk of patients over-reporting perceived improvements in symptom severity.

## 5. Conclusions

Overall, the results from this study are positive and suggest that PTNS is a minimally invasive, non-surgical, non-hormonal, and effective third-line treatment for OAB in an Australian population. These results suggest that PTNS may be a second-line treatment for patients with OAB not responding to conservative management or for patients aiming to avoid surgical approaches. This study should promote future investigation into the long-term maintenance of the therapeutic benefit of PTNS in the Australian setting, particularly looking at patient demographics to find out who experienced symptomatic improvements, and possibly a comparative study between the benefits and cost of PTNS and surgical treatments, such as intradetrusor injections.

## Figures and Tables

**Table 1:** Improvements in OAB symptoms from Phase 1 and Phase 2 (paired *t*-test).

		Mean Pre-Treatment Symptom Severity (0–4) ± SD	Mean Post-Treatment Symptom Severity (0–4) ± SD	Improvement	*p*-Value
Urinary Frequency					
	Phase 1 (n = 164)	1.41 ± 1.2 *	0.94 ± 1.1 *	0.48 (33.8%) *	<0.05
	Phase 2 (n = 64)	1.31 ± 1.2 *	0.57 ± 0.7 *	0.74 (56.5%) *	<0.05
Urinary Urgency					
	Phase 1 (n = 160)	2.48 ± 1.0 *	1.74 ± 1.0 *	0.74 (29.8%) *	<0.05
	Phase 2 (n = 47)	1.83 ± 0.9	1.68 ± 1.1	0.15 (8.0%)	=0.40
Nocturia					
	Phase 1 (n = 166)	1.74 ± 1.1 *	1.22 ± 1.0 *	0.52 (29.8%) *	<0.05
	Phase 2 (n = 51)	1.31 ± 0.9	1.06 ± 1.0	0.25 (19.0%)	0.05
Urinary Urge Incontinence					
	Phase 1 (n = 160)	2.06 ± 1.2 *	1.42 ± 1.0 *	0.64 (31.0%) *	<0.05
	Phase 2 (n = 47)	2.09 ± 1.1	1.38 ± 0.9	0.71 (34.0%)	=0.47

* denotes statistically significant result.

## Data Availability

The access date to the data is 5 May 2023.

## Appendix A

ICIQ-OAB Scoring system pdf/document.

## Appendix B

PTNS methods as per Michael R van Balken [28].
